# *Lamium* Plants—A Comprehensive Review on Health Benefits and Biological Activities

**DOI:** 10.3390/molecules24101913

**Published:** 2019-05-17

**Authors:** Bahare Salehi, Lorene Armstrong, Antonio Rescigno, Balakyz Yeskaliyeva, Gulnaz Seitimova, Ahmet Beyatli, Jugreet Sharmeen, Mohamad Fawzi Mahomoodally, Farukh Sharopov, Alessandra Durazzo, Massimo Lucarini, Antonello Santini, Ludovico Abenavoli, Raffaele Capasso, Javad Sharifi-Rad

**Affiliations:** 1 Student Research Committee, School of Medicine, Bam University of Medical Sciences, Bam 44340847, Iran; bahar.salehi007@gmail.com; 2 Department of Pharmaceutical Sciences, State University of Ponta Grossa, Ponta Grossa, Paraná 84030900, Brasil; lorenearmstrong@hotmail.com; 3 Department of Biomedical Sciences, University of Cagliari, s.s. 554 bivio Sestu, I-09042 Monserrato, CA, Italy; 4 Faculty of Chemistry and Chemical Technology, Al-Farabi Kazakh National University, Almaty 480012, Kazakhstan; balakyz@mail.ru (B.Y.); sitigulnaz@mail.ru (G.S.); 5 Department of Medicinal and Aromatic Plants, University of Health Sciences, 34668 Istanbul, Turkey; ahmet.beyatli@sbu.edu.tr; 6 Department of Health Sciences; Faculty of Science, University of Mauritius, Réduit 80837, Mauritius; sharmeenjugs@gmail.com; 7 Department of Pharmaceutical Technology, Avicenna Tajik State Medical University, Rudaki 139, Dushanbe 734003, Tajikistan; 8 CREA-Research Centre for Food and Nutrition, Via Ardeatina 546, 00178 Rome, Italy; alessandra.durazzo@crea.gov.it (A.D.); massimo.lucarini@crea.gov.it (M.L.); 9 Department of Pharmacy, University of Napoli Federico II, Via D. Montesano, 49-80131 Napoli, Italy; 10 Department of Health Sciences, University Magna Graecia, viale Europa-Germaneto, 88100 Catanzaro, Italy; 11 Department of Agricultural Sciences, University of Naples Federico II, 80055 Portici, Italy; rafcapas@unina.it; 12 Zabol Medicinal Plants Research Center, Zabol University of Medical Sciences, Zabol 61615-585, Iran

**Keywords:** *Lamium* plants, antiviral, antioxidant, anti-inflammatory, cytotoxicity

## Abstract

This work is an updated snapshot of *Lamium* plants and their biological activities. The main features of the plant are described and the components of its essential oils are summarized. The traditional medicinal uses of *Lamium* plants has been reported. The presence of these chemicals i.e., hydroxycinnamic acids, iridoids, secoiridoids, flavonoids, anthocyanins, phenylpropanoids, phytoecdysteroids, benzoxazinoids, betaine can provide biological activities. After the discussion of antioxidant properties documented for *Lamium* plants, the biological activities, studied using in vitro models, antimicrobial, antiviral, anti-inflammatory, anti-nociceptive activity, and pain therapy and cytotoxicity and cytoprotective activity are here described and discussed. Finally, targeted examples of in vivo studies are reported.

## 1. Introduction

Medicinal plants have been used since antiquity to treat illness and discomfort; their knowledge has been propagated from generation to generation by indigenous and local populations instigating the ethnobotanical study. Considering their global use, traditional, therapeutic and industrial value, many natural products are being investigated to lead the production of new drugs, wherefore, families and genera of plants with great potential are being researched for this purpose [1,2,3]. The recent review of Durazzo et al. [4] gives a current picture of main features of botanicals, by describing the strict relationship between the main plant biologically activity compounds and the nutraceutical role of botanicals [4,5,6,7,8,9,10].

*Lamiaceae* is a widespread family of flowering plants, also known as the mint family [11]. It is well distributed around the continents and, has about 250 genera described, which have 7,852 accepted species names [12]. The largest genera are *Salvia*, *Scutellaria*, *Stachys*, *Plectranthus*, *Hyptis*, *Teucrium*, *Vitex*, *Thymus* and *Nepeta* [3,11,13]. The plants of this family are normally shrubs or herbs with aromatic compounds in their leaves or flowers, such as essential oils. Many species are cultivated for their medicinal properties like antiseptic, antispasmodic, calmative, antimicrobials and, it is also used for culinary, fragrance, flavor and aromatherapy [11,14,15,16].

Within the family *Lamiaceae*, the genus *Lamium* is herbaceous and possess annuals or perennials forms, comprising around 40 species found in temperate and subtropical regions of Africa, Asia and Europe [16,17,18,19]. Regarding the botanical aspects, the leaves are cordate or reniform, ovate to lanceolate with an acute apex and cordate base, being petiolate on the lower nodes and sessile or uncommon amplexicaule at the upper nodes. It presents inflorescences with verticillasters in the axils of the floral leaves (2-12 flowered). The calyx is campanulate or tubular with subequal teeth. The corolla is bilabiate and shows a dark purple, yellowish green, white, yellow, etc. [17,19]. Species well studied of the genus are: *L. album* L., *L. purpureum* L., and *L. maculatum.* The popular name of “dead nettle” is due to the superficial similarity of the stinging nettles and, they do not have trichomes which can release toxic compounds [20].

The genus is used in folk medicine as antispasmodic, astringent, anti-proliferative, anti-inflammatory, antiviral, regulatory for sebaceous secretions and, also for the treatment of hypertension, scrofula, paralysis, prostate, menorrhagia, uterine hemorrhage, leucorrhea, trauma, fracture [19,20,21,22].

Ecologically this genus shows important characteristics such as self-pollination, being hosting for different insects species and they attract bumblebee queens and honeybees, which represents the entomophilous pollination [19]. This focused review wants to give an updated snapshot of main beneficial properties of *Lamium* plants, with a focus on antimicrobial, antiviral, anti-inflammatory, anti-nociceptive activity, and pain therapy and cytotoxicity and cytoprotective activity, in order to better address nutraceutical uses, formulations and applications; therefore, this review aims to bring information found in the literature data about different aspects of the *Lamium* genus and to demonstrate its importance regarding traditional, medicinal, chemical composition, biological and pharmacological activities, as well as industrial potential and economic use. The search was carried out using search engines Scopus, Science Direct, and PubMed and the following keywords were typed: *Lamium* and health benefits; *Lamium* and biological activities; *Lamium* and antioxidant properties; *Lamium* and antiviral activity; *Lamium* and anti-inflammatory activity; *Lamium* and antimicrobial activity; *Lamium* and antischistosomal activity; *Lamium* and antinociceptive activity; *Lamium* and pain therapy; *Lamium* and cytotoxicity; *Lamium* and cytoprotective activity; *Lamium* and anti-tyrosinase activity; *Lamium* and anticancer activity; *Lamium* and in vitro studies; *Lamium* and in vivo studies; *Lamium* and clinical studies.

## 2. A Shot on the Biologically Active Compounds in *Lamium* Plants

Chemically, *Lamium* is distinguished by the presence of different classes of chemical constituents, which we can mention hydroxycinnamic acids, terpenoids, among them iridoids and secoiridoids, flavonoids, anthocyanins, phenylpropanoids, phytoecdysteroids, benzoxazinoids, betaine [20,21,22]. Thus, the presence of these chemicals can provide biological activities tested in vitro and in vivo assays, such as antioxidant, anti-inflammatory, antimicrobial, antischistosomal, for pain relief in rheumatism and arthritis, a tonic for constipation, antinociceptive, anticancer [16,20,21,22]. Most of the bioactivities of *Lamium* species are linked to their principal constituents, that is, phenolics and essential oils [23,24,25,26]. Polyphenols, flavonoids, terpenes, steroidal derivatives, and ecdysteroids account for various biological activities of this species of *Lamium* [27,28,29,30,31]. In this regard, it is worth mentioning the review of Carović-Stanko et al. [32] focused on the *Lamiaceae* species and their secondary metabolites encompassing a wide array of beneficial functions and their applicability as sources of functional foods.

### 2.1. The Phenolic and Terpenoid Compounds of Lamium Plants

**Figure 1:**
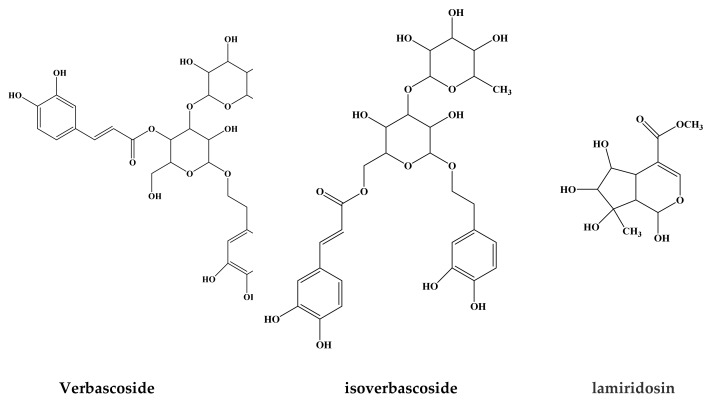
The chemical structure of some biologically active molecules from *Lamium* species.

Here some studies showing the isolation of phenolic and terpenoid compounds are reported. For instance, Deng et al. [33], by studying constituents in the herb of *Lamium maculatum* L. var Kansuense, isolated and characterized ten compounds were obtained and they were identified as d-mannitol, beta-sitosterol, stigmasterol, rutin, 3′-methylquercetin-3-*O*-rutinoside, n-butyl-beta-D-fructopyranoside, daucosterol, acteoside, 20-hydroxyecdysone, allantoin. Nugroho et al. [34] isolated and characterized two new flavanol glycosides, together with other three just known using column chromatography from aerial parts of *L. amplexicaule*. The Czerwinska et al. [22] reported that the main constituents of the aerial part of *L. Album* were phenylpropanoids, iridoids, flavonoids and phenolic acids. Czerwinska et al. [35] in the herb of *L. album* underlined phenylpropanoid glycosides along with iridoids and flavonoids might be the valuable bioactive compounds present in these species. The peculiar constituents of *Lamium* plants are phenlypropanoids and iridoids. The phenylpropanoids consist in a group of compounds with a great diversity of structures including simple phenylpropanoids (i.e., derivatives of cinnamyl alcohols, cinnamic acids, phenylpropanes) and complex phenylpropanoids [36]. Generally they can occur rarely in the free form and are often present in bound form, linked to carbohydrates, as examples of main forms found in *Lamium* plants. Beside phenylpropanoids, another main constituent and potential chemotaxonomic marker of *Lamium* plants are iridoids: they are monoterpenes consisting of a cyclopenta[c]pyranoid skeleton (iridane skeleton) and mainly occurring as glycosides [37]. Molecules such as verbascoside, cis-acteoside, and lamalboside (lamiusides A), lamiusides B, C, and E, lamiridoside, lamiol, caryoptoside, albosides A and B are examples of phenylpropanoid glycosides, described *Lamium album* L. [21,27,35,38,39]. For instance, in the aerial parts of the *L. album* plant two phenylpropanoids glycosides, verbascoside and isoverbascoside (Figure 1), can make up 55% of the total phenolic compounds measured [40]. The iridoids found in the genus can work as markers of *L. album*, *L. amplexicaule*, *L. garganicum*, *L. maculatum*, and *L. purpureum* [20].

### 2.2. Essential Oil Constituents of Lamium Plants

Considering how the therapeutic importance of *Lamiaceae* species is mostly based on their volatile oils [41], here essential oil constituents of *Lamium* plants are described. Even though plants belonging to the Lamiaceae family are recognized to contain an eminent amount of essential oils, the plants from the genus *Lamium* of the subfamily Lamioideae possess a small number of essential oils. The yields of the essential oils extracted from the fresh flowers of *Lamium* plants differ between 0.01–0.31% [42].

Numerous authors have studied the flavor composition from *Lamium* species [42,43,44]. However, the volatile components of only some species have been well studied so far, out of the 25 species of *Lamium* that are accepted [12]. Various studies have demonstrated that the biological and chemical activities of essentials oils from *Lamiaceae* plants, not merely varied within the species and its varieties but even amid the different agro-climatic and geographical regions [45,46,47,48,49]. Such variations in the composition of *Lamiaceae* plant essential oils can be essentially related to genetic as well as environmental factors that also define the genetic expressions and thereby influence the chemical constituents of the oils [50]. A study by Alipieva et al. [43] on the essential oils of four *Lamium* species from Bulgaria (namely, *L. album*, *L. purpureum*, *L. garganicum*, and *L. maculatum* flowers) collected from nine natural populations were analyzed by using GC-MS. Although a similarity of the volatile profiles of all samples was observed, quantitative and qualitative variations in oil composition of the plants obtained from different locations were also seen. For instance, hexahydrofarnesyl acetone was observed in *L. maculatum* (at Losen mountain: 1.9% while at Ljaskovetz: 1.2%), *L. purpureum* (at Vlado Trichkov: 1%) and *L. album* (at Vlado Trichkov: 0.2%) though not from all location sites. Besides, predominantly in *L. maculatum* and *L. garganicum* were identified other terpenoids, such as β-caryophyllene and α-humulene, found in highest concentrations in *L. maculatum* (at Losen mountain: 1.6% and 0.8%). Table 1 depicts more such examples where variations in the composition and percentages of main constituents of essential oils of *Lamium* species grown in different regions and/or countries.

Furthermore, the study of Flamini and workers [42] showed that the composition of essential oils varied, in different organs of *Lamium* species investigated. For instance, in *L. purpureum*, the content of α- and β-pinene was 35.7% in the leaves, 7.5% in the bracts while it was 75.3% in the flowers. In addition, in *L. bifidum*, bracts and leaves displayed a profile characterized by the common presence of germacrene D, β-caryophyllene, α-humulene, and β-elemene, while contrastingly, the flowers emitted profiles revealing high percentages of myrcene (47.2%), β-caryophyllene (11.8%) and sabinene (11.0%).

**Figure 2:**
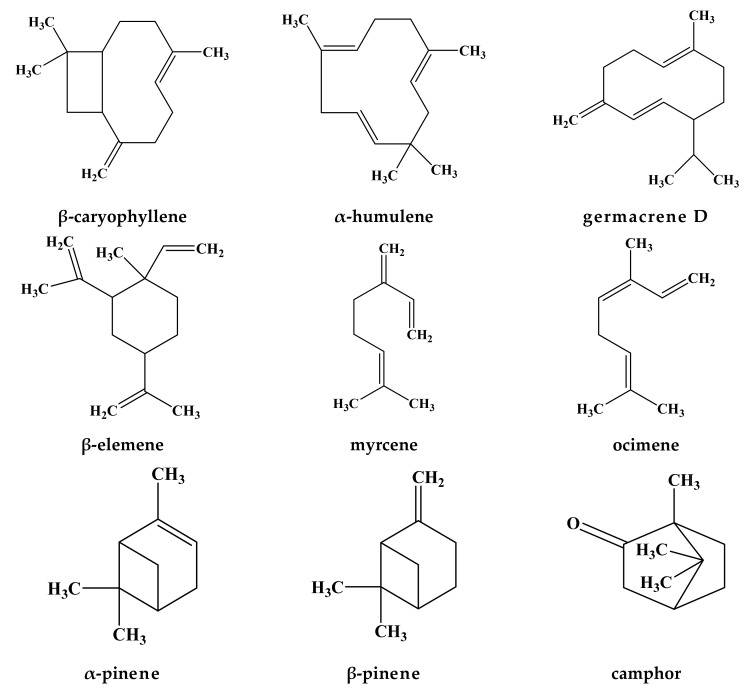

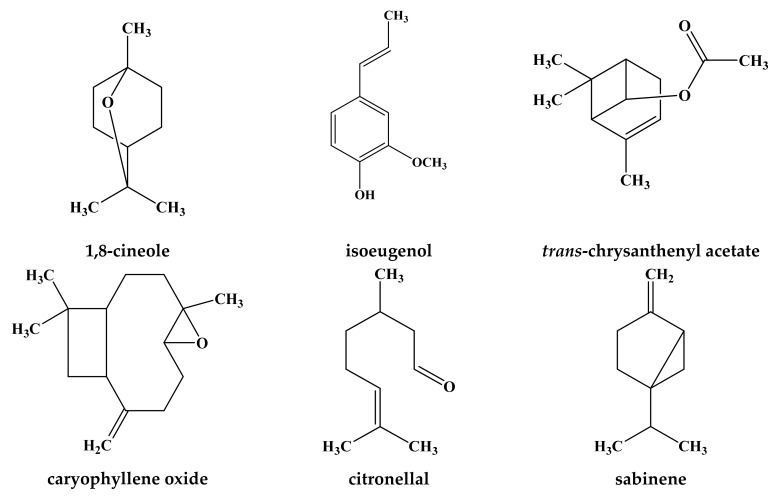
The chemical structure of the main volatile secondary metabolites from *Lamium* species.

The previous work of Kapchina-Toteva et al. [51] also investigated the effect of micropropagation on the essential oil content in leaf extracts of *Lamium album* L. plants grown under in vitro and ex vitro conditions in contrast to in situ-grown plants. The in situ-grown plants were found to consist of 45 hydrocarbons, and as a result of micropropagation, the content of these compounds reduced about two-fold, reaching 24 during in vitro cultivation, and finally 19 after ex vitro acclimation. Moreover, while the in situ plants were characterized by the accumulation of long-chain alkanes (nonadecane; heneicosane; heptadecane; tricosane), both in vitro and ex vitro plants contained shorter hydrocarbons such as octane (3.9% and 1.1%, respectively) and undecane (3.0% in in vitro samples). Besides, certain alcohols such as phytol; octen-3-ol; 3-hexene-1-ol and acetate were observed to rise in micropropagated plants compared to in situ-grown ones. As for terpenes, 60 compounds were detected in the oil of in situ plants while it decreased in both in vitro and ex vitro ones—44 and 35 compounds. A variation in the terpene composition was also detected. The major sesquiterpenes in the in situ-grown plants were germacrene D (6.9%) and β-caryophyllene E (1.1%). In the course of micropropagation, elevated accumulation of these compounds was observed in both, in vitro- and ex vitro-grown plants [germacrene D (44.1% and 46.7%, correspondingly) and β-caryophyllene E (13.0% and 6.5%, correspondingly)]. Therefore, these results demonstrated the significance of growing conditions which were accountable for the variability in the level and composition of plant metabolites. The chemical structure of the main volatile secondary metabolites from *Lamium* species are represented in Figure 2.

## 3. Traditional Medicinal Uses of *Lamium* Plants

Plants have been used by a human as food and medicine since ancient times. Examples of the traditional medicinal uses of *Lamium* plants are here described. White dead-nettle (*Lamium album* L.) used for decades in Europe, China, and Japan during times of famine [65]. Different aerial parts of this plant are edible and traditionally used as raw or cooked food in some countries. Especially in some dishes in Mediterranean and neighboring areas [66]. In addition, non-stinging nettle is considered a base component of some well-known vegetarian dishes and salads [40]. *L. album*, when added to food supplements, can prevent menstrual, musculoskeletal disorders and ameliorate fat metabolism [67,68]. *L. amplexicaule* is used in the preparation of Japanese traditional rice porridge which called “seven spring herbs” [69].

In traditional and folk medicine worldwide some *Lamium* species used in the treatment of fracture, hypertension, leucorrhoea, paralysis, putrescence, trauma and some gynecological diseases like menorrhagia, uterine hemorrhage, vaginal and cervical inflammation, bleeding after childbirth and believed to be a contraceptive, etc. [70,71,72]. Ethnobotanical studies indicate the use of aerial parts and floral branches of *L. album* for different kidney problems like the exertion of stones [73,74]. Leaves decoction and infusion for respiratory tract problems [75].

Moreover, there are many types of activities, e.g., antipyretic, astringent, bronchitis, diuretic, emollient, expectorant, insomnia, pains, sciatica, vasodilator, hemostatic, wound healing, antihypertensive, anti-inflammatory which recorded by literature [76,77,78,79,80]. *L. amplexicaule* used as anti-rheumatic, laxative and diaphoretic [81]. Sometimes fresh leaves of *L. amplexicaule* are crushed a paste formed used topically to joints swelling [82]. Aerial parts decoction of *L. galeobdolon* traditionally used for fever, malaria, warts, constipation, hair loss, rheumatism, dandruff, hemorrhage [83,84] depression, nerve tonic [85].

## 4. Biological Activities *Lamium* Plants

Our mental and physical well-being is directly related to what we introduce with the diet. In fact, the nutritional content of what we eat affects the composition of our cell membranes, blood, tissues, organs, skin, and so on. It has long been a common opinion, widely shared also by the scientific world, that diet and nutrition are also important factors that can, depending on the case, protect us or promote pathological conditions and chronic diseases. From this perspective, the great variety of biologically active molecules present in plants can offer a general condition of ‘protection’ and play an important role in maintaining a state of well-being of the individual.

In the years 1970–1980, scholars of popular culture and history of medicine began to record the traditional health habits of the peoples in the various countries, with an approach that tried to capture the full breadth without expressing value judgments. In this context, the notion of folk medicine has extended its meaning since the boundaries between popular traditions and scientific evidence present undeniable differences. Over time, therefore, the research has tried to find scientific evidence to validate or refute practices in the profane medical culture.

Folk medicine has made extensive use of plants of the *Lamium* genus over the centuries. The most common uses are described in the countries of the Mediterranean basin (Europe and North Africa) and in Western Asia. Buds, leaves, and flowers are also widely used in the kitchen for the preparation of various recipes of local tradition in many countries of the Mediterranean basin [21]. From health benefits, plants of the *Lamium* genus have found widespread applications in folk medicine thanks to the large presence of chemical compounds that constitute effective active ingredients in many situations of therapeutic interest.

### 4.1. In Vitro Studies

Most of the scientific evidence on the *Lamium* genus has been conducted through in vitro studies over the past 15–20 years. Extracts from the shoots, leaves, and flowers, have shown many biological activities. In this section, particularly, we will review the species of the *Lamium* genus used about various medicinal uses, highlighting those most promising studies given a possible use of the active ingredients extracted from these plants.

#### 4.1.1. Antioxidant Activity

As is known, antioxidant molecules can be technically defined as agents that prevent or slow down the phenomenon of oxidation. Reactive oxygen species (ROS) are unavoidable sub-products of cellular aerobic metabolism [86]. However, free radicals can also originate from prolonged exposure to UV rays, cigarette smoke, and air pollution. The reactive oxygen molecules are capable of damaging the structures of the cell through the establishment of so-called oxidative stress, a situation that, if not kept under control, can lead or exacerbate many pathological states [87]. A large number of molecules, so-called antioxidants, can interrupt the chain of radical reactions and thus prevent damage to cells [88].

Our body can counteract the activity of free radicals through endogenous antioxidant mechanisms and the introduction through the diet of exogenous substances [89]. Among the endogenous factors are enzymes such as superoxide dismutase (SOD; EC 1.15.1.1) which catalyzes the dismutation of the superoxide radical (O_2_^−^) into either molecular oxygen (O_2_) or hydrogen peroxide (H_2_O_2_), catalase (CAT; EC 1.11.1.6), which removes intracellular H_2_O_2_, and reduced glutathione (GSH) [90]. Among the non-exogenous non-enzymatic substances with antioxidant properties, we recall Vitamin E, Vitamin C, carotenoids, polyphenols, and anthocyanins instead.

While many foods have a protective effect on free radicals, on the other, improper eating habits can increase their activity, for example, a diet too rich in animal fats, excessive consumption of some vegetable oils and fatty fish, excess of iron, food intolerances.

The antioxidant properties are determined in vitro by common assays: the trolox equivalent antioxidant capacity (TEAC), oxygen radical absorbance capacity (ORAC), total radical-trapping antioxidant parameter (TRAP), ferric-reducing antioxidant power (FRAP) and 2,2-diphenyl-1-picrylhydrazyl (DPPH) radical scavenging activity assay [91,92]. They vary in their principles, mechanisms, and experimental condition, in the reference compounds used, i.e., trolox, gallic acid, or catechins and in how endpoints are measured [93,94,95].

These methods are based on the transfer of electrons (ET) or the transfer of hydrogen atoms (HAT) [96,97]. Another antioxidant mechanism is the transition metal chelation, (TMC): transition metals ions may be chelated by polyphenols, leading to stable complexed compounds [98]. The determination of the antioxidant power should be measured with at least two or three assays [99,100]. A more in-depth and correct investigation must also include the determination of the phenolic content and that of the flavonoids.

As marked by several authors [101,102,103] in the procedure of determination of antioxidant properties three critical elements should be taken into account: the extraction procedure, the antioxidant capacity measurements and the expression of results.

In this regard it is worth mentioning the point of view of Durazzo and Lucarini, [7] that well summarized the actual main strategies of research for evaluating antioxidant properties: the evaluation of bioactivities of pure compounds and/or their mixtures; the study of different biologically active compound-rich extracts and how these fractions contribute to the activity of total food extract; the isolation of extractable and non-extractable compounds.

The assessment of the interaction of bioactive compounds as antioxidant properties represents the first step for the evaluation of the health properties of medicinal plants [4]. Many species belonging to the *Lamium* genus have been studied to identify their antioxidant properties. Carović-Stanko et al. [32] pointed out that most of the *Lamiaceae* sources of antioxidants belong to the subfamily Nepetoideae, such as basil, lemon balm, marjoram, mint, oregano, rosemary, sage, etc [104]. *Lamium album* L. is absolutely the most studied species. Trouillas et al. [105] have studied and compared the water-soluble fraction of 16 plants, including *L. album*, typical of Limousin, a central-southern region of France. Presumably, the whole plant was used, whose main constituents are acid phenols, flavonoids, mucilage, iridoids. Water-soluble extracts have been tested for the ability to inhibit the DPPH radical, the superoxide radical generated by the xanthine/xanthine oxidase (X/XO) system, and the inhibition of the hydroxyl radical generated by the Fenton reaction. It is interesting to note that in the first two cases (DPPH and X/XO) the extracts of *L. album* (non-stinging nettle) showed to be very effective when compared with the other species studied; particularly, it was 12-16 times more effective than extracts of *Urtica dioica* (stinging nettle). This result diverged, significantly, from that obtained with a methanol extract of *L. album* that showed the same ability to inhibit the DPPH radical at an extract concentration about 140 times lower [106]. This discrepancy could be due to the better ability of methanol to extract flavonoids (≈193 mg of gallic acid equivalents per g of extract (mg GAE/g)). This higher extraction capacity was corroborated by the greater amounts of phenolic compounds extracted when compared to extraction in water. The content of phenolic compounds of the methanol extract was almost five times greater than the aqueous extract. This result was in agreement with the content of phenolic compounds found in methanol extracts of specimens of another species of the *Lamium* genus, *L. amplexicaule* L. collected during the flowering period in a region of south-eastern Anatolia, Turkey (≈184 mg GAE/g) [107]. The presence of polyphenols could, moreover, be the determining factor in explaining why the methanol extract and hexane of *L. amplexicaule* showed a significant reduction in the formation of nicked DNA and increased the native form of plasmid DNA pBR322.

Hydroxyl radicals generated by the Fenton reaction are known to cause oxidatively induced breaks in DNA strands via the subsequent free radical-induced reaction on plasmid DNA. Hydroxyl radicals can react with nitrogenous bases of DNA producing base radicals and sugar radicals. Polyphenols are potential protecting agents against the lethal effects of oxidative stress and offer protection to DNA by chelating redox-active transition metal ions [108,109]. For instance, Yumrutas et al. [107] showed that *L. amplexicaule* hexane extract seemed to possess a greater ability to protect DNA than the methanol extract. Hence, it might be said that available non-polar compounds in the hexane extracts might be contributing to phenolic compounds for protecting DNA.

As another example, the antioxidant effect of butanol extracts from wild specimens *L. album* and *L. purpureum* L. (red dead nettle) collected in Romania were compared for DPPH and chemiluminescence activity. A possible correlation between the chemical composition, especially for the amount of total phenols, and the antioxidant activity of the extracts was found [110]. In both cases, the extracts possessed dose-dependent scavenger activity evaluated after 30 min. Incubation with extracts, in all dose levels tested, whereas, the *L. purpureum* extract (1% concentration) exhibited the highest scavenging activity compared with *L. album* extract.

Vladimir-Knežević et al. [111], by studying different medicinal plants of the Lamiaceae family such as *Salvia officinalis*, *Mentha longifolia*, *Melissa officinalis*, *Lavandula angustifolia*, *Satureja montana* concluded that *Lamiaceae* species are a rich source of various natural AChE inhibitors and antioxidants.

Danila et al. [112] aimed at assessing the phenolic content of *L. album* and *L. maculatum* methanolic extracts, and their antioxidant capacity: for the DPPH assay the EC_50_ (µg/mL) values were 32.3 ± 0.1 for *L. maculatum* extract and 63.5 ± 0.7 for *L. album* extract, while in the ABTS assay EC50 (µg/mL) values were 13.2 ± 0.1 for *L. maculatum* extract and 19.9 ± 0.5 for *L. album* extract.

On the other hand, many studies have proven that some natural antioxidants are a double-edged sword. They can, under certain conditions, act as pro-oxidants in vitro, triggering lipid peroxidation, DNA damage and apoptotic phenomena [113]. Phenolics and carotenoids can also exhibit prooxidant activities, mainly in the presence of redox-active transition metal ions [114,115,116].

#### 4.1.2. Antiviral Activity

As is known, viruses can replicate only within a host cell, exploiting their metabolic apparatus and using their own genetic information; however, multiplication occurs only in cells susceptible to the virus, that is, provided with specific superficial receptors and able to perform the replicative phases of its genome.

The search for an antiviral compound must be based on the interaction of the drug with specific stages of viral replication; for example, it can act on cellular penetration of the virus, on the replication of its genome, on protein synthesis or on the release of new viruses from the host cell. Herbal medicines and purified natural products provide a rich resource for novel antiviral drugs [117].

Some chemical compounds have been isolated in plants of the *Lamium* genus having interesting antiviral activities that are here reported. It is worth mentioning a phytochemical study of the aqueous extract of the flowering tops of *L. album*, a component herb in a commercial liver health herbal formula, that led to the identification of the antiviral activity of some iridoids [118]. Isomers lamiridosins A and B were found to significantly inhibit hepatitis C virus entry in vitro showing an IC_50_ 2.31 μM. Interestingly, the parent iridoid glucosides demonstrated no anti-HCV entry activity.

#### 4.1.3. Antimicrobial Activity

Generally, the term ‘natural antibiotics’ refers to those substances endowed with antibacterial activity deriving from plants. In fact, antibiotics of natural origin do not derive only from plants, but also from fungi, bacteria, and animals. Antibiotics are substances used to fight bacterial infections and may have bacteriostatic action (i.e., inhibit bacterial growth) or bactericidal (i.e., they can kill bacteria). A similar argument can be made for the antimycotics that are used to counteract the development of pathogenic fungi.

Some types of plants can produce antibacterial and antifungal substances, even if they present an activity, usually, much lower than that possessed by antibiotics deriving from fungi and bacteria [119]. Furthermore, it is good to remember that the antibacterial or antifungal substances contained in these plants can interfere with possible pharmacological treatments already in place. Plants also contain other compounds that could potentially be hazardous to health. However, in the popular medicine of emerging countries, the use of plant preparations to counteract the growth of pathogenic microorganisms is often described.

Antifungal activity of *L. tenuiflorum* Fisch and Mey against some medical yeast species was described by Dulger et al. [120]. The ethanol extracts obtained from the leaves, rootstock, and the combined formulation of Turkish endemic specimens have been investigated for their antifungal activities against medical yeast *Candida* and *Cryptococcus* species. The extract (in the form of sticky black substances) was dissolved in DMSO before testing. The combination of plant extracts (1:1 ratio) was used in this test. Comparing the obtained result with those of the antifungal drug ketoconazole used as a reference, it was noted that the combination of plant extracts (both leaves and rootstock) exhibited greater antifungal effect against *Candida albicans* and that *Candida* spp. was more susceptible than *Cryptococcus* spp. Unfortunately, in that study, there is no correlation between quantity and type of polyphenols present in the extract of *L. tenuiflorum* and the antimycotic activity found. Conversely, none of the extracts obtained from *L. galactophyllum* Boiss and Reuter, *L. macrodon* Boiss and Huet and *L. amplexicaule* displayed activity towards *Candida albicans* ATCC 10231 [121]. Lacking such an antimycotic power could be due to the different quantity of polyphenols contained in these species, ranging between 94 and 112 mg GAE/g in respect to higher values found for *L. album* and *L. amplexicaule* which extracts ranged between 184 and 193 mg GAE/g [106,107].

The great variety of species of the *Lamium* genus present in Turkey [121] has meant that traditional folk medicine made extensive use of *Lamium* herbs. On the contrary, *L. album* extracts possess little antifungal activity as found by Chipeva et al. [122] who evaluate the antimicrobial activity of *L. album* plants. The extracts, obtained from leaves and flowers, were harvested either in the wild or from in vitro propagated plants. Four solvents (chloroform, methanol, ethanol, and water) and two methods of extraction (Sоxhlet, thermostat) were used. The different combinations of extraction solvent and extraction method led to results that also varied due to the origin of the plant, i.e., wild or in vitro propagated. In conclusion, *L. album* extracts possessed a broad spectrum of antibacterial activity with greater efficacy towards Gram-positive bacteria. However, as the extracts have not been chemically characterized it is impossible to determine which molecule may be more responsible for such an antibacterial activity.

#### 4.1.4. Anti-Inflammatory, Anti-Nociceptive Activity, and Pain Therapy

Arachidonic acid is the main precursor of eicosanoids, substances involved in the body’s inflammatory response. In the presence of tissue damage, enzymes belonging to the phospholipase class A2 release the arachidonic acid from the membrane phospholipids. From this, two different molecular types can be obtained: the series 2 of prostaglandins and thromboxanes (from the cyclooxygenase pathway) and the series of leukotrienes (from the lipoxygenase pathway). The synthesis of the series 2 of prostaglandins (PG2) and thromboxanes is mediated by the enzyme cyclooxygenase, which is present in the human organism in the form of COX1 and COX2. The synthesis of leukotrienes is linked to the activity of the enzyme 5-lipoxygenase. From these observations, pharmacological research on molecules able to counteract the effects of arachidonic acid derivatives by inhibiting the enzymes involved in the inflammatory cascade has started. To this end, enzymes extracted from both animal and vegetable sources used as enzymatic model systems to evaluate the anti-inflammatory effect of plant extracts and active ingredients are also used.

Aqueous extracts of *L. album* have been shown to inhibit lipoxygenase activity in vitro [105], at relatively low concentrations of extract (IC_50_ ≈1.5 mg/mL). As these extracts have not been further characterized, it is not possible to attribute this inhibition activity of lipoxygenase to a specific molecule. It is very likely that this effect is due to more than one molecule and through several mechanisms. In fact, many studies have established that free oxygen radicals are implicated in inflammatory processes [123] and that phenolic compounds can block lipoxygenase activity, or they can function as scavengers of free radicals which are released during the inflammatory cascade of arachidonic acid [124]. Anti-inflammatory bioactivity of compounds was also proved with aqueous-methanolic extract of *L. album* herb in human neutrophils as recently showed by Czerwinska [35]. This effect appears to be due to the inhibition of the release of some inflammation mediators, such as the IL-8 and 3 TNF cytokines, by neutrophil granulocytes.

Rheumatoid arthritis is a multifactorial chronic, systemic and disabling inflammatory disease with an undefined etiology, but probably of autoimmune origin. It mainly affects the symmetrical joints, but also tendons, synovium, muscles, bags and other tissues of the organism. Rheumatoid arthritis develops because, in a genetically predisposed subject, an environmental triggering event activates an auto-immune response; there is thus an abnormal activation of the immune system, which affects the joints causing chronic inflammation and consequent joint damage.

Rheumatoid arthritis can also be fought with plants [125] whose extracts can be used at least to reduce the quantities of methotrexate, one of the elective drugs used in the treatment of this pathology. As for the *Lamium* genus, the evidence that some species can provide benefits in the treatment of rheumatoid arthritis is quite weak [126] and are mostly speculative and based on the antioxidant and anti-inflammatory properties of *L. album* extracts.

About the inflammatory activity of *L. album* extracts, cannot be ignored a study in which the leaves of this herb have been used as a placebo to test the anti-inflammatory activity of *U. dioica* (stinging nettle) [127]. That study prompted from the observation that the sting of the common stinging nettle has long been used for self-treatment of arthritic pain. A randomized controlled double-blind crossover study in twenty-seven patients with osteoarthritic pain at the base of the thumb or index finger was performed to ascertain if the daily application of nettle leaves in the painful area brought relief. *L. album* leaves were chosen as a placebo since leaves are almost indistinguishable from stinging nettle leaves. It was remarkable that after a week of treatment the score reduction with *U. dioica* leaves was significantly greater than that with placebo (*L. album* leaves).

Pain therapy aims to recognize, evaluate and treat chronic pain in the most appropriate way. There are several classes of drugs that can be used for the treatment of pain. The type of drug to be used can vary depending on the origin, the nature and the intensity of the painful stimulus that is intended to be treated. Depending on the circumstances can be used: non-steroidal anti-inflammatory drugs (e.g., ketoprofen, diclofenac, naproxen and nimesulide), opioid analgesics (e.g., codeine, tramadol, buprenorphine, fentanyl, oxycodone, methadone, hydromorphone and morphine), antidepressants, very useful in the treatment of neuropathic pain (e.g., amitriptyline, clomipramine, duloxetine); anticonvulsants, also useful in the treatment of neuropathic pain (e.g., gabapentin, pregabalin); local anesthetics (e.g., lidocaine). Unfortunately, the use of such drugs is often accompanied by sometimes serious side effects, which may sometimes outweigh the benefits of the drug. For this reason, over the past few years, general attention has shifted to non-pharmacological therapies (e.g., radiotherapy, cryotherapy, thermotherapy, massages, physiotherapy, relaxation techniques). Increasing attention has also concerned the active ingredients from medicinal plants. Although widely used in traditional folk medicine, there are only a few studies investigating the potential analgesic effects of the *Lamium* genus. Most of the studies have been carried out concerning different genera of the *Lamiaceae* family, but very few are those concerning the *Lamium* genus [128].

#### 4.1.5. Cytotoxicity and Cytoprotective Activity

Cytotoxicity is the measurement of how much a substance can damage or kill cells. This measurement can be performed both in vitro and in vivo, and this difference is significant, since it is one thing to measure the cytotoxic activity of a chemical agent on a cell culture in a homogeneous medium (e.g., a fibroblast layer on a medium of culture) and the measurement of cell *viability* in vivo is very different, where many biochemical and other factors are involved. Most of the cytotoxicity studies of plant extracts have been conducted in vitro because it is the simplest method. In vitro studies, in turn, offer several advantages; e.g., they are highly reproducible simplified systems, they allow to analyze the cellular and molecular mechanisms of toxicity, the identification of early damages and allow to contain costs and get rapid responses concerning animal experimentation. The main criticism that can be found in systems for measuring cytotoxicity in vitro concerns the excessive simplification of such methods concerning a multicellular organism. Nevertheless, in vitro tests are widely used; the cytotoxicity can be carried out and evaluated with different essays. The most used is the MTT assay, the SRB assay (with Sulforhodamine B), the TB assay (with Trypan blue). In the study of cytotoxicity mechanism, the choice of the cellular model should respond to the need to study in detail the organ-specific effects of certain compounds or mechanisms of action in specific cell types. Unfortunately, most often the cellular model is chosen independently of the needs mentioned above; for this reason, the results obtained in the different studies are hardly comparable.

As instance, Veleva et al. [129] studied the changes in the functional characteristics of tumor and normal cells after treatment with extracts of white dead-nettle, by adhesion test, MTT (3-(4,5-dimethylthiazol-2-yl)-2-5-diphenyl tetrazolium bromide), transepithelial resistance (TER), immunofluorescence staining and trypan blue exclusion test: extracts from *L. album* L. change TER and actin filaments, and somehow may block cell mechanisms, leading to the polarization of MDCK II cells (Madin-Darby canine kidney cells II).

From another work [105], aqueous extracts of the whole *L. album* plant have been shown to induce cytotoxicity in the mouse tumor cell line, the B16 mouse melanoma cells at relatively low concentrations. It is difficult to determine what may be the causes of this cytotoxicity although it could be related to the content of phenolic compounds of the extract [105].

Polyphenols can act as antioxidants through various mechanisms, including hydrogen donating reactions, metal chelation, inhibition of cytochrome P450 isoforms and up-regulation or protection of antioxidant defenses (e.g., intracellular glutathione levels) [130]. In particular, the possible cytotoxicity of these phenylpropanoids has long been debated. For instance, verbascoside has shown increased chromosome aberrations and in vitro sister chromatid exchanges in human lymphocyte cultures [131]. However, the results may be due to instability and degradation of verbascoside in caffeic acid and 3,4-dihydroxyphenyl ethanol [131]. The genotoxicity of verbascoside seems to have been ruled out entirely by a recent study [132]. This study clearly demonstrates that diets rich in verbascoside do not give rise to any mutagenic activity, resulting in non-cytotoxic to animals and suggesting its possible use in both animal and human diets.

In fact, products based on dried leaves and flowers of *L. album* are already on the market. *L. album* is numbered among dermatological plants with anti-inflammatory activity, and this plant is also used in wound healing [21]. Skin fibroblasts proliferation is considered as the most important initial stage of tissue repair. Thus, Paduch et al. [133] analyzed the plant extracts activity on human skin fibroblasts (HSF) proliferation and viability in order to add information on the effectiveness of these products. The sensitivity of HSF cells in culture to methanol, ethyl acetate, and heptane extracts of *Lamii albi flos* were investigated. Extracts with methanol, ethyl acetate and heptane of *Lamii albi flos* were prepared by heating 20 g of plant material with 300 mL of the appropriate solvent for 5 h at a temperature of 60 °C at reflux. Each of these extracts was subsequently concentrated under reduced pressure at 30 °C up to a volume of 100 mL. The extracts thus obtained were used for the determination of flavonoids, pentacyclic triterpenes, and iridoids. The triterpene component seemed to be responsible for the absence of cytotoxicity of the heptane extracts even at high concentrations and, indeed, triterpenes can exert stimulatory effects on the proliferative capacity of HSF cells.

When a model of chemical stress induced by potassium dichromate in human hepatoblastoma HepG2 cells was used, ethanolic extracts of *L. album* showed a cytoprotective effect in vitro [40]. Purified extract counteracted ROS formation in oxidative stress conditions in tested cells. The cytoprotective effect of 50 µg/mL *L. album* purified ethanolic extract seems related to the presence of verbascoside, which exhibited the highest cytoprotective action from all the polyphenols identified in the ethanolic extract.

It is interesting to reiterate that solvent extracts of the same species, *L. album*, can exert cytoprotective or cytotoxic effects according to the methods of extraction, application of the extract and the tested cell lines. Moscova-Doumanova et al. [134] investigated the effect of methanol, and chloroform extracts, obtained from in vivo and in vitro cultivated plants of *L. album*, on the cell viability, adhesion, and cell cycle of the type A549 human lung cell line. Different combinations of methanol and chloroform extracts were tested. Preliminary results showed that both the extracts have a cytotoxic effect on lung cancer cells. They caused a reduction in the adhesion properties of the cells with a stronger effect by extracts from in vivo plants. After 48 h of incubation time, all extracts cause retention in the G2 phase while a mixture of them leads to the apoptosis. However, without characterization of the extracts, it is not possible to hypothesize the molecular mechanism of the observed phenomena.

Other applications on corneal disease are described as follows. The cornea is the membrane that covers the front of the eye, through which it is possible to glimpse the iris and the pupil. Transparent and avascular, this structure represents the first ‘lens’ that the light encounters in its path to the brain. The cornea is, in fact, an essential element of the ocular dioptric system: it allows the passage of light rays towards the internal structures of the eye and helps to focus the images on the retina. The cornea’s optical function is carried out thanks to its perfect transparency and the regularity of the contact surface with the air. Therefore, any inflammation and damage to the corneal epithelial should be quickly eliminated to maintain corneal transparency. In this context, the powerful antioxidant and anti-inflammatory properties of *L. album* extracts above described could make, in perspective, this medical herb a promising candidate in the formulation of natural remedies for topical use in corneal diseases.

This prompted Paduch et al. [135] to evaluate the effect of *L. album* extract on human corneal epithelial cells (10.014 pRSV-T cell line) cultured in vitro. In that study, the first goal achieved was to ascertain the ethanol extract of *L. album* was non-toxic to human corneal epithelial cells at concentrations up to 125 μg/mL. Ethanol extract contained polar compounds which contribute to maintaining cells intact, or even, stimulate cellular mitochondrial metabolism as verified by MTT assay. Besides, flavonoids and polyphenolic compounds, better represented in ethanol extracts, also contributed to the reduction of inflammatory phenomena and ROS scavenging. Therefore, it cannot be excluded that soon, after further in vivo experiments, supplements for the treatment of mild eye diseases based on extracts of *L. album* herb may be used.

**Figure 3:**
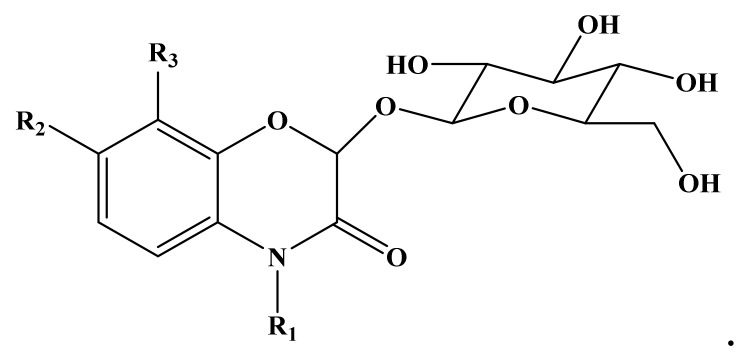
Benzoxazinone glucoside skeleton.

At this point, it is worth mention *L. galeobdolon* L., commonly known as the ‘yellow archangel, that has good potential as an ingredient for the preparation of functional foods. This species is a wildflower widespread in Europe and has been introduced elsewhere as a garden plant. An ethnobotanical study carried out on 49 edible wild plants traditionally harvested and consumed in a region of the Basque Country, Northern Spain, described the recreational use by children of nectar sucked from the base of *L. galeobdolon* flowers. Different benzoxazinoids (BXs), present as glucosides (Figure 3), have been identified in the yellow archangel.

These compounds represent a class of indole-derived plant metabolites that work in defense against numerous parasites and pathogens [136]. Many recent studies have reported antimicrobial, anticancer, reproductive stimulatory effects, system stimulators central nervous system, and reduction of appetite and weight of BXs derivatives and their derivatives [137].

#### 4.1.6. Antityrosinase Activity

Tyrosinase (EC 1.14.18.1) is a ubiquitous enzyme containing two copper ions. The dinuclear copper center of tyrosinase catalyzes the o-hydroxylation of monophenols, oxidation of catechols [138,139], quinonization of dihydroxycoumarins [140], o-aminophenols and aromatic o-diamines [141]. Tyrosinase is also one of the key enzymes in melanin biosynthesis. In animals, as the enzyme catalyzes the first two main steps of the melanogenesis. Overproduction of melanin can result in various hyperpigmentation disorders including melasma and age spots. Thus the discovery of new tyrosinase inhibitors has been since many years the main goal of numerous investigations [142,143]. Many studies deal with chemical compounds extracted from plants. Even though the presence of phenolic compounds in plants of the genus *Lamium* is abundant, and potentially there are many phenolic compounds able to influence the activity of tyrosinase, there are very few studies that describe such an influence on the tyrosinase enzyme.

Nugroho et al. [34] reported how two flavanol glycosides in methanol extracts of *L. amplexicaule* showed in vitro inhibitory activity against the mushroom tyrosinase. The tyrosinase inhibition mechanism and related kinetics have not been studied in detail. In the study, it is hypothesized that the high group of hydroxyl groups present on the isolated flavonoid molecule may be responsible for binding to the catalytic site of tyrosinase, and result in an inhibition phenomenon according to previous studies [144,145].

The current study of Etsassala et al. [146] applied a fast screening method using a cyclic voltammetry technique for evaluating anti-tyrosinase activity of twenty-five species of plants from the *Lamiaceae* family: among these, those that showed a fast current inhibition rate at a minimum concentration when compared to a kojic acid standard were classified as having the greatest anti-tyrosinase activity such as *Salvia chamelaeagnea*, *S. dolomitica*, *Plectranthus ecklonii*, *P. namaensis*, and *P. zuluensis*.

### 4.2. In Vivo Studies

The observation of in vivo phenomena is often considered more relevant to factual reality than in vitro reproductions, although the latter is equally useful because they allow analyzing a single phenomenon, isolating it from the context that could create a background noise that is too high to be able to distinguish the phenomenon clearly.

Unfortunately, in vivo studies using plants of the *Lamium* genus are very few compared to in vitro studies.

An in-depth study evaluated the anti-inflammatory and antinociceptive activities of various extracts prepared with methanol, dichloromethane, n-butanol, and water from the aerial parts of some species of the *Lamium* genus [25]. In this study, conducted on male Swiss albino mice, extracts of *L. eriocephalum* subsp. *eriocephalum*, *L. garganicum* subsp. *laevigatum*, *L. garganicum* subsp. *pulchrum* and *L. purpureum* var. *purpureum* were administered to the animals to alleviate inflammatory pain in a model of ear edema and in carrageenan-induced and Prostaglandin E_2_-induced hind paw edema. The experimental data demonstrated that *L. garganicum* subsp. *laevigatum* and *L. garganicum* subsp. *pulchrum* displayed remarkable anti-inflammatory and antinociceptive activities in mice at 200 mg/kg dose without inducing any gastric damage.

The biological activities of the herbal extracts of the genus *Lamium* described up to now have concerned organic solvent extracts or water extracts. These extracts allowed to obtain concentrated solutions of phenolic compounds, flavonoids, iridoids, terpenes, steroidal derivatives, enriched with the various components depending on the polarity of the extracting mixture.

An unusual extraction procedure has been described in a study of the biological activity of an *L. album* oil extract [147] by using a biphasic solvent system consisting of 70% ethanol and sunflower oil at a ratio of 1:1, after the maceration of above-ground of *L. album* in water. The oil extract (OE) was then studied in models of hemolytic anemia (HA) induced with intramuscular (IM) administration of phenylhydrazide chloride to white mongrel male rats. Three groups of animals were formed: (i) intact animals, (ii) negative control group (with induced hemolytic anemia but untreated, (iii) OE treated (with induced hemolytic anemia and treated). The results showed that the administration of extract had anti-anemic effects since all blood parameters (e.g., number of erythrocytes, hemoglobin levels, hematocrit, and red blood cell indices) were significantly better throughout the twelve weeks of the experiment. The authors suggested that the anti-anemic effect of OE may be due to the antioxidant action of chlorophyll preparation. This explanation is not fully convincing since the sunflower oil used to obtain OE contains on average 59% of linoleic acid [148]. Recently, linoleic acid has been proved to induce red blood cells and hemoglobin damage via an oxidative mechanism, eventually leading to partial acute anemia [149]. In other words, if chlorophyll had effectively carried out anti-anemic action, such an action would be even stronger as it would have contrasted not only the anemic effect due to the administration of phenylhydrazide chloride, but also that induced by the linoleic acid contained in OE. It would have been interesting to know the effect of the administration of the extractor oil only in a further control group, but this eventuality has not been taken into consideration by the authors of this study.

An interesting in vivo use of *L. amplexicaule* has been described in Punjab, a region of southern Pakistan. In this region, the leaves of *L. amplexicaule* are administered orally to ruminants affected by helminth infections, at a rate of 250 g at a time [150]. The duration of treatment is not standardized and is not performed by veterinary staff but by local pastors. The efficacy of the treatment, therefore, is not certified by veterinarians and needs further investigation carried out with criteria and procedures of the veterinary medicine. However, the wide use of *L. amplexicaule* as an anthelmintic in ruminants suggests that there are concrete possibilities for effective action by substances contained in the plant.

### 4.3. Clinical Studies

Randomized controlled trial (RCT) is a study in which people are allocated at random (by chance alone) to receive one of several clinical interventions. If the in vivo studies conducted with herbs belonging to the *Lamium* genus are few, the RCTs studies are even less. Below we give an account of the few RCTs we have come to know.

Atopic dermatitis (AD) is a pruritic, chronic and inflammatory skin disease, the onset of which often coincides with the pediatric age. The ‘atopic’ appellation, attributed to dermatitis, underlines the absence of a skin location. Shapira et al. [151] describe a brief report about forty-nine patients who were recruited for a two weeks treatment to test the efficacy of tri-herbal combination on AD in a randomized, placebo-controlled trial. *L. album* was one of these three herbs. The medication was taken orally three times daily for two weeks. That study found that tri-herbal combination induced a highly significant improvement in both objective and subjective parameters of AD. However, placebo treatment induced equally positive results in all measured aspects, so it is not possible to attribute any therapeutic effect to the medicament *L. album* containing.

## 5. Conclusions and Future Perspectives

Beside specific aspects of plant biochemistry, this review underlined the use of *Lamiun* plants (some species) in the formulation of natural remedies for topical use, preparation of functional foods, possible action as tyrosinase inhibitors, among others. Lamiaceae is one of the most extensive and diverse plant families about their ethnomedicinal properties. Besides the great representativity of species, chemotaxonomic markers are individuated as well as targeted biological functions.

Generally, the ancient science of phytotherapy study represents a great challenge for future research in pharmacological and medical fields: from the exploitation of chemistry of plants to intervention studies until clinical trials in humans. New frontiers should be based on an integrated and multidisciplinary approach of research, in terms of health benefits and sustainable health applications and addressed towards advanced technologies such as the nanotechnologies and chemometrics.

## Figures and Tables

**Table 1:** Main components of essential oil from *Lamium* species studied from the year 1976–2018.

Plant Name	Place/Country of Collection	Parts Used	Extraction Method Used	% Yield	Main Components	References
**(1) *Lamium amplexicaule* L.**	Khorassan-e Razavi province in northeastern Iran	aerial parts (flowers and leaves) *	Hydro-distillation	0.1 (*w*/*w*)	Trans-phytol (44.8%), octadecanol (12.0%), hexadecanoic acid (11.8%) and hexahydrofarnesyl acetone (10.6%)	[52]
	Huntsville, Alabama, USA	aerial parts **	Hydro-distillation	NA	Germacrene D (18.5–34.9%), (E)-caryophyllene (2.5–11.9%), α-pinene (2.2–16.2%), β-pinene (2.0–10.6%) and 1-octen-3-ol (3.5–8.0%)	[53]
	Pergole, Arcidosso Municipality, South Tuscany, Italy	Flowering aerial parts	Hydro-distillation	NA	Trans-chrysanthenyl acetate (41.1%), germacrene D (28.9%) and α-pinene (6.8%), ocimene (0.8%)	[42]
	Northeast of Tehran, Iran	aerial parts *	Hydro-distillation	0.1(v/w)	Germacrene-D (22.3%) and camphor (18.1%)	[54]
	El Dakahlyia governorate, Egypt	Leaves	NA	NA	Isophytol (14.8%), 9,12,15-ocadecanoic acid methyl ester (19.2%), 6,10,14-trimethyl-2-pentadecanone (8.0%), dibutyl phathalate (6.1%), nonacosane (5.5%), hexadecanoic acid (3.4%) and nonyl phenol (3.2%)	[55]
	Syria	NA	Dry evaporation	NA	Imedazol and pyrimidene	[56]
						
	NA	NA	NA	0.09	NA	[57]
**(2) *Lamium purpureum* L.**	Huntsville, Alabama, USA	aerial parts **	Hydro-distillation	NA	Germacrene D (15.0–46.3%), α-pinene (4.1–15.3%), β-pinene (6.3–16.3%), and 1-octen-3-ol (4.2–15.3%), β-elemene (3.7–16.0%)	[53]
	Pergole (Arcidosso Municipality, South Tuscany, Italy	Flowering aerial parts	Hydro-distillation	NA	Germacrene D (35.4%), β-pinene (26.8%) and α-pinene (13.4%), ocimene (2.9%)	[42]
	Japan	Aerial parts	Steam distillation	NA	1-Octen-3-ol, cis-3-hexen-1-ol, phenethyl alcohol, benzyl alcohol, phenol, 0-, m-, and p-cresols, guaiacol, eugenol	[44]
**(3) *Lamium maculatum* L.**	Experimental station of Faculty of Pharmacy, Zagazig University, Egypt	aerial parts **	Hydro-distillaion	0.35 (v/w)	β-caryophyllene (14.8%), caryophyllene oxide (13.8%), Z,E-α-franesene (10.1%), dihydroedulan I (9.13%), α-humulene (6.1%), bornyl formate (6.0%) and α-bisabolene (5.3%)	[58]
	NA	Leaves	NA	NA	Hexahydrofarnesylacetone (22%)	[59]
**(4) *Lamium hybridum* Vill**	Pergole (Arcidosso Municipality, South Tuscany, Italy	Flowering aerial parts	Hydro-distillation	NA	Germacrene D (39.0%), (Z)-ocimene (8.7%), methyl salicylate (7.5%) and β-caryophyllene (6.1%), ocimene (11.6%)	[42]
**(5) *Lamium bifidum* Cyr.**	Pergole (Arcidosso Municipality, South Tuscany, Italy	Flowering aerial parts	Hydro-distillation	NA	Germacrene D (34.9%), sabinene (12.4%), β-caryophyllene (11.5%), α-humulene (6.8%)	[42]
**(6) *Lamium garganicum* L. subsp. *laevigatum* Arcangeli**	Athens, Greece	aerial parts ***	Hydro-distillation	0.31	1,8-cineole (47.5%), citronellal (25.1%) and isoeugenol (11.8%)	[60]
**(7) *Lamium album* L.**	Behshahr, Mazandaran Province, North of Iran	Flowering aerial parts	Hydro-distillation	0.2 (*w*/*w*)	6,10,14-trimethyl-2-pentadecanone (10.2%) and 4-hydroxy-4-methyl-2-pentanone (9.1%)	[61]
	Experimental field of the Kaunas Botanical Garden of Vytautas Magnus University, Lithuania	Plants in the vegetation period	Supercritical carbon dioxide extraction method	NA	Prenol, farnesene-beta- E, tridecanol n, dodecanoic acid n, hexadecane-n, squalene, tetradecanol-n, undecane–n, benzoate-isopentyl, dodecanoate -butyl, phytone, neophytadiene	[62]
	NA	Aerial part	NA	0.04–0.46	NA	[57]
	NA	Flowers	NA	0.05	NA	[57]
	Kharkiv region, Ukraine	Leaves	NA	NA	α-Terpeniol, linalool, squalene, spatulenol, α-Bisabolol	[63]
**(8) *Lamium moschatum* Mill.**	NA	Flowers	Steam-washed	NA	Caryophyllene	[64]
**(9) *Lamium striatum* Sibth. et. Smith**		Flowers	Steam-washed	NA	Carboxylic acids	[64]

NA—Not Available/applicable; * Air-dried; ** fresh; *** fresh air-dried.
